# Supplementary material for: Food demand displaced by global refugee migration influences water use in already water stressed countries
Source: Nat Commun. 2023 May 23;14:2706. doi: 10.1038/s41467-023-38117-0 (PMC10205736; doi:10.1038/s41467-023-38117-0)
Supplement: Supplementary file 1 — Supplementary Information [file 41467_2023_38117_MOESM1_ESM.pdf]

Supplementary Information for

Food demand displaced by global refugee migration influences water use in already water stressed countries

Leonardo Bertassello, Marc F. Müller, Adam Wiechman, Gopal Penny, Marta Tuninetti, Michele Müller-Itten

Corresponding Author Marc F. Müller.
E-mail: mmuller1@nd.edu

This PDF file includes:

Figs. S1 to S8
Tables S1 to S8
References for SI reference citations

Notation We use upper and lower case variables to represent absolute and per capita quantities, respectively. Variables in prime notation ($\cdot'$) are used to represent quantities of food (tonnes) to distinguish them from volumes of virtual water (m^3). Subscript are used to indicate the type of food (k), the country of production of the food (p) and the countries of origin (o) and destination (d) of the refugees. Superscript y indicates the considered year in the 2005-2016 period.

A. Origin Matrix of Traded Virtual Water. Consider a network of N countries described by M_{pdk}, a $N \times N$ matrix indicating the virtual water fluxes $[m^3]$ associated with the trade of a good of type k from country p to country d. M has an empty diagonal. Then, $1 \times N$ vectors E and I indicating the total virtual water flux exported from and imported to each country are expressed:

$$E_{pk} = \sum_{d} M_{pdk} \qquad \text{[S-1]}$$

$$I_{dk} = \sum_{p} M_{pdk} \qquad \text{[S-2]}$$

Let the $1 \times N$ vector P indicate the water footprint of production in each country. Then the $1 \times N$ vector of total flux F of virtual water transiting through each node can be described as the $1 \times N$ vector:

$$F = C + E = P + I \qquad \text{[S-3]}$$

where C is a $1 \times N$ vector indicating the virtual water consumed in each country.

We want to determine the matrix X_{pdk} $[-]$, which is the fraction of all virtual water associated with good type k that was consumed in country d that was produced (extracted) in country p. A key assumption in our approach is that all virtual water fluxes leaving a given node have the same composition in terms of their (original) source country, regardless of the destination country. In other words, all outgoing fluxes from node d to nodes p (export and consumption) will have the same X_{pdk} for a given commodity. A helpful analogy would be a situation where each node produces a slightly different 'flavor' of the goods of type k. This assumption implies perfect trade openness whereby no preference is given for locally produced goods. The matrix X_{pdk} then represents the concentration of the subset of goods k that were produced in node p, within the broader flux of goods k leaving node d. Accordingly, we express the mass balance at node d of the goods of type k that were produced in node p:

$$X_{pdk} F_{dk} = P_{dk} \mathbf{1}_{d=p} + \sum_{i} X_{pik} M_{idk} \qquad \text{[S-4]}$$

Recall that the diagonal of M is zero. The left hand side of the equation represents the total flow out of node d of the of goods of type k that were produced in node p. It is obtained by multiplying total outflows F_{dk} by the fraction X_{pdk} which represents the share of goods produced in p. The right hand side represents total flow into node d of goods k produced at node p. It is obtained by summing fluxes M_{idk} coming into node d from all other nodes i; appropriately weighing each by the concentration of goods k produced in p (X_{pik}). For the node that produces the good (i.e. $p = d$), a production term is added.

Omitting the subscript k to simplify notation, S-4 can be written in matrix form:

$$X \mathsf{diag}(F) = \mathsf{diag}(P) + XM \qquad \text{[S-5]}$$

where $\mathsf{diag}\,(F)$ and $\mathsf{diag}\,(P)$ represents a diagonal matrix with the vectors F and P as diagonal terms. Solving for X, we get

$$X = \mathsf{diag}(P)(\mathsf{diag}(F) + M)^{-1} \qquad \text{[S-6]}$$

In the numerical implementation, we exclude all nodes that do not participate in the trade of the considered good (i.e. we exclude the nodes i for which $F_i = 0$). Because these nodes do not take part in the market, the corresponding row vectors of origin X_i are designated as NA. The matrix $\boldsymbol{X}$ $[\frac{m^3 \text{VW in country p}}{m^3 \text{VW in country d}}]$ can be used to determine where the virtual water consumed in a given country is ultimately coming from.

B. Per capita water footprint of food. The CWASI dataset (1) combines FAO trade data with a biophysical model of food production. Unlike previous water footprint estimates, this dataset is temporally disaggregated (i.e. there are annual water footprint and virtual water estimates), based on the assumption the variability of virtual water fluxes is driven by variations in crop yields and traded volumes, rather than climate (evapotranspiration) variability (2), which is still indirectly included in crop yields. By using the yield as a proxy variable for water footprint evaluations, the dataset accounts for technological improvements, for instance pertaining to crop varieties, fertilizers or irrigation techniques. It should be noted, however, that crop evapotranspiration is kept constant at a value equal to the average of the 1996-2005 period. This approximation was evaluated in (2) for maize, rice, soy, and wheat, and found to introduce an uncertainty of approximately 10% to water footprint estimates

The CWASI dataset provides:

- Matrices $M^{(y)}_{pdg}$ of fluxes of virtual water associated with the trade of commodity g between countries p and d during year y

- Vectors P_{pg} of virtual water associated with the production of raw commodities g in country p during year y. Note that the production goods g are *raw* commodities (e.g., wheat) and there is not a one-to-one correspondence with *traded* commodities (e.g., Maccaroni) in the matrices $M^{(y)}_{pdg}$

Note that the superscript (y) is omitted in the following derivation to simplify notation, but the annual time scale of the CWASI database allows for annual estimation of virtual water contents.

We reconcile raw and traded commodities g by aggregating them into *types* (or 'family') of goods k, where traded commodities are associated with the raw commodity that dominates their composition. For example, bread, flour, maccaroni and wheat are aggregated into one type (or 'family') of goods k:

$$M_{pdk} = \sum_g M_{pdg} \quad \text{[S-7]}$$

$$P_{pk} = \sum_g P_{pg} \quad \text{[S-8]}$$

The water footprint of consumption of good type k in country d can then be expressed as:

$$C_{dk} = P_{dk} + \sum_i M_{idk} - \sum_i M_{dik}$$

where the terms of the right hand side represent the production, import and export of good type k in country d.

In effect, we conduct a simple mass-balance of the virtual water fluxes entering (production and imports) and exiting (exports) at the country-level (d) and good type (or 'family') level (k). Accordingly, it is possible for a country to have a negative per capita virtual water consumption if for instance, a country imports and/or produces a raw commodity with low virtual water footprint and then heavily exports a derived commodity with a high virtual water footprint. In this way, the country is virtually exporting its water resources through trade of the given family of good. However, on any given year of the study period, such negative virtual water links account for less than 5% of per capita water footprints for at least 120 of the 131 considered countries (Table S7). Similarly, on any given year, those countries that have negative virtual water links totalling more than 5% of per capita blue water footprint host for 6% or less of total global refugees (Table S7). This suggest that negative virtual water fluxes arising from the production of processed good is unlikely to have a substantial effect on our results.

We then compute the unit footprint of consumption of good type k in country d by normalizing by the total consumption C' (in tonnes) of that good in that country. This information is obtained from national Food Balance sheets from the Food and Agriculture Organization (FAO) as described in the following section:

$$W_{dk} = \frac{C_{dk}}{C'_{dk}}$$

We finally compute the virtual water associated with the consumption of food type k in country d of a person with average dietary habits from country o:

$$c_{odk} = W_{dk} c'_{ok}$$

where c'_{ok} corresponds to the per capita consumption (in tonne) of food type k in country o, also obtained from the FAO food balance sheet (see following section). Note that the virtual water associated with the consumption of food type k by a native person in country d is a special case of the above Equation (i.e. the country of diet o is identical to the country d where the food is consumed):

$$c_{ddk} = W_{dk} c'_{dk}$$

Finally, we can use the matrix X_{pdk} from Section A to distribute the virtual water volume c_{odk} across the production countries p where that water has been extracted:

$$c_{odkp} = c_{odk} X_{pdk}$$

In the above Equation, c_{odkp} represent the annual virtual water volume extracted in country p, for the consumption of good type k in country d by a person with an average diet from country o.

C. Food consumption data. For each type of goods k, the FAO Food Balance dataset contains total annual production P'_{ok}, exports E'_{ok}, I'_{ok}, changes in stock $\Delta S'_{ok}$, and domestic supply quantity Z'_{ok}, defined as the mass balance of the production, trade, and stock flows. Note that the $(\cdot)'$ differentiates these variables, in [tonnes], from the corresponding variables reported in volume units of virtual water in the CWASI dataset. As before, upper and lower case variables indicate absolute and per capita quantities, respectively.

As the FAO Food Balance dataset notes, this total consumption represents many forms of consumption, including feed, food, seed, processing, losses, and other uses. Our analysis focuses the refugee water footprint on food consumption dynamics, so we extracted the fraction of this consumption attributed to food. Following (3), we compute the fraction Φ_{ok} of good type k consumed in country o attributed to food as,

$$\Phi_{ok} = \left[\frac{C'_{ok}}{Z'_{ok}}\right]_{1/2} \quad \text{[S-9]}$$

Variable H'_{ok} indicates the FAO Food Balance reported tonnes of food of type k consumed in country o. To address reporting outliers in the dataset, after computing Φ for each year, we used the 2005-2016 median (indicated as the operator $[\cdot]_{1/2}$) of Φ for each type of goods k in country o. We then use mass balance consideration to estimate the per capita total consumption in a country o for good type k:

$$c'_{ok} = \Phi_{ok}\frac{P'_{ok} - E'_{ok} + I'_{ok} - \Delta S'_{ok}}{\mathsf{Pop}_o} = \Phi_{ok}\frac{Z'_{ok}}{\mathsf{Pop}_o} \qquad \text{[S-10]}$$

Two substantive challenges emerged when attempting to reconcile the FAO food balance dataset with the dataset of virtual water fluxes, which is based on FAO trade and production data:

- First, the FAO Food Balance already reports demand and production/trade flows at a good type level (e.g., wheat and products). Several of these groups do not have corresponding types in the FAO production datasets, on which the CWASI virtual water dataset (1) is based. When possible, we combined orphan FAO Food Balance groups with their "parent" types: for instance, soybean oil was combined with the soybean family. In rare circumstances, the orphan FAO Food Balance group was altogether removed. For instance, since the CWASI virtual water database does not have data for all fermented beverages included in the FAO Food Balance, we did not consider this group. Overall, our analysis examined 62 commodity types, and their associated goods and sub-Food Balance groups (if applicable) are displayed in Table S-4. This rearrangement was accounted for when computing Φ and c'. For Φ, we (i) computed Φ for each FAO Food Balance group and then (ii) took a weighted mean of these estimates by (FAO trade) commodity type) (k), using proportion of the (FAO trade) family-level consumption represented by each FAO Food Balance group. For c', we summed the c' values for each FAO Food Balance group with each a commodity type.

- Unfortunately, the FAO Food Balance dataset does not have entries for three important countries in global refugee dynamics during our 2005-2016 window: Syria, Somalia, and the Democratic Republic of the Congo (DRC). For these three countries, we constructed the domestic supply quantity (Z'_{ok}) using the detailed trade matrix provided by the FAO (and openly available at https://www.fao.org/faostat/en/#data/TM). Guided by the FAO Food Balance's group definitions, we aggregated trade and production data for all commodities g in a given FAO Food Balance group k to estimate Z' for each country o as:

$$Z'_{ok} \approx \sum_g P'_{og} + \sum_g I'_{og} - \sum_g E'_{og} \qquad \text{[S-11]}$$

Note that this approximation neglects interannual variations in stocks ($\Delta S'$) which are not reported in the FAO trade dataset.

To estimate Φ, we first filtered out types of good (which, recall, combine both raw and transformed commodities) that are likely not traded by the considered country. These types of goods were attributed a value of $\Phi \approx 0$ and were identified as either (i) having a Z' below an (arbitrary) threshold of 100 metric tons per year or (ii) having $\Phi > 0$ for only one or fewer countries of the considered region (e.g., Western Asia for Syria). For the remaining types, Φ was obtained as the weighted geometric mean of its value observed in a set of comparable countries. This set of prediction countries, and the corresponding weights, were estimated using a linear regression framework applied to a set of 15 nutritional outcomes assembled for 2016 in all countries (including Syria, Somalia and the DRC) by the Global Nutrition Report. The estimation strategies hinges on the assumption that the set of countries and weights that most accurately predict these 15 nutritional outcomes for Syria (or respectively Somalia or the DRC) with also be the most accurate predictor of Φ in that country. For Syria, this assumption was formalized in a multiplicative model where we regress all observed nutritional outcomes (N=15) in Syria, against their value for K comparable countries:

$$\log X_{SYR} = \alpha_0 + \alpha_1 \log X_1 + \alpha_2 \log X_2 + ... + \alpha_K \log X_K + \epsilon \qquad \text{[S-12]}$$

where the vector X_{SYR} represents the 15 nutritional outcomes from Global Nutrition Report for Syria, and vectors $X_1, ...X_K$ the corresponding values for predictor countries 1 to K. Weights α_k are the weights corresponding to each country of the prediction set (plus the intercept α_0) obtained through ordinary least squares, with ϵ an independent and identically distributed error term. The regression in Equation S-13 was estimated for all permutations of prediction countries drawn from the same subregion as the country with missing Φ. For example, for Syria, this subregion consisted of the 16 countries of the global nutrition report located in Western Asia and the regression was run for all possible permutations of subsets of these countries of sizes 2 to 16. The weights (α_k) and prediction set corresponding to the regression with the most information (i.e. the lowest Bayes Information Criterion – BIC –, which penalizes model complexity) was retained to predict Φ as:

$$log\Phi_{SYR} \approx \alpha_0 + \sum_{k=1}^{K} \alpha_k \Phi_k$$

where Φ_k are the values of Φ in the K countries of the regression in Equation S-13 with the lowest BIC value, and α_k are the corresponding regression coefficients.

Consumption is finally estimated by combining approximations of Φ and Z' for the missing countries:

$$c'_{ok} \approx \frac{Z'_{ok}\Phi_{ok}}{\mathsf{Pop}_o} \qquad \text{[S-13]}$$

The approach was evaluated through cross validation, where values of Φ and Z' were estimated in a set of countries that (i) are adjacent to countries with missing observations (Syria, the DRC and Somalia) and (ii) Φ and Z' were available from the FAO food balance sheet. For each validation country, we computed the weighted absolute percent error (WAPE) across food types as:

$$WAPE_o = w_{ko}\frac{1}{Y}\sum_y \mid \frac{\tilde{X}_{ok} - X_{ok} \mid}{X_{ok}}$$

where $\tilde{X}^{(y)}_{dk}$ and $X^{(y)}_{dk}$ are the predicted and observed characteristics (Z' or Φ) associated with good type k in country j on year y; Y is the total number of years in the considered period and $|\cdot|$ is the absolute value operator. Weights w_{ok} are given by

$$w_{ok} = \frac{Z'_{ok}\Phi_{ok}}{\sum_k Z'_{ok}\Phi_{ok}}$$

and represent the proportion of each family of good k in the country's total food consumption. Their purpose is to down-weight errors on good types that make up negligible portions of the country's total food consumption. Results on Table S5 show (WAPE) on the order of 30% in the validation countries for both Φ and Z'.

D. Usable flow increase of the Yarmouk River. We evaluated the increase in the Yarmouk transboundary Yarmouk streamflow from Syria into Jordan during the portion of our study period that corresponds to the Syrian civil war. To do so, we computed the difference between annual Yarmouk flow volumes observed at Al Wehda Dam on the Jordan-Syria border (see 4) and flow average in the 2006-2010 period, before the Syrian conflict.

We estimated the portion of the flow increase that is attributable to abandonment of irrigated agriculture in upstream Syria using the "no-conflict" counterfactual scenario constructed in (4, Figure S4). The estimate includes an uncertainty range that was constructed in (4) by taking the upper and lower bounds in the determination of crop water use from reservoir storage. The upper and lower bounds, respectively, neglect irrigation return flows or neglect the contribution of subsurface water to summer discharge (which is assumed to be entirely formed of irrigation return flow).

We then sought to determine the proportion of that refugee-related flow increase that was likely captured for irrigation in downstream Jordan. Using the comprehensive model of the Jordanian Water sector in (5), we computed the increase in the annual water used by Jordan Valley Authority (JVA), which manages irrigation water on the Jordanian side of the Jordan Valley. The JVA records annual irrigation water used in three distinct sectors (North, Middle and South) along the King Abdullah Canal that diverts the Yarmouk river downstream of Al Wehda. We considered three combinations of sectors (North, North+Middle and North + Middle + South) to provide lower, middle and upper bounds estimates of increased water uses. The North sector alone likely does not use up all the increase in the Yarmouk flow and so is an underestimate, whereas considering all three zones likely also includes provision from other water sources (e.g., groundwater) and so overestimate reliance on Yarmouk water. Each year, we divided the obtained water use estimates for each combination of sectors by the observed increase in the Yarmouk flow. We then multiplied these fractions by the portion of Yarmouk flow increases that are attributable to refugees to get the flow increase that is both associated with refugee migration and also actually captured by Jordan. We combined upper and lower bounds on both flow attribution and JVA capture to estimate the uncertainty range displayed on Figure 3A. The above procedure assumes that increases in JVA allocations in 2011-2015 are due to increased Yarmouk flow and not to increased groundwater use of provision from Tiberias lake, both of which also supply the King Abdullah Canal. Data from the comprehensive model in (5) show no substantial difference in Lake Tiberias diversions between the 2006-2010 and 2011-2015 periods.

E. Pareto-frontier of refugee resettlement plans. After estimating the (inverse) average individual hardship resolution rate λ_d (as described in the main document) and the per capita marginal increase in water stress $\Delta s_{d,\mathsf{SR}}$, we proceed to the Pareto-Optimization of resettlement plans. Let the vector $\mathbf{x}$ represent a resettlement plan, with each term representing the number Q_d of refugees resettled out of each considered country d. The Pareto optimization considers the trade-off between:

(i) The aggregate relief of the individual hardship of refugees. Assuming that hardship is exponentially distributed across the refugees of each country d, this corresponds to:

$$\Delta\tau(\mathbf{Q}) = \sum_d \int_0^{Q_d} -\frac{1}{\lambda_d}\ln\frac{x}{R_d}dx \qquad \text{[S-14]}$$

(ii) The aggregate relief of the water stress of current countries of refuge:

$$\Delta S(\mathbf{Q}) = \sum_d Q_d \Delta s_{d,\mathsf{SR}} \qquad \text{[S-15]}$$

The Pareto frontier could then be obtained numerically through brute force by generating random instances of $\mathbf{Q}$ and taking the convex hull of the corresponding $(\Delta\tau, \Delta S)$ pairs.

For a more direct estimation, consider that the Pareto frontier results from the combined optimization of the two criteria of the tradeoff for different combinations of weight assigned to each criterion. This can be encoded in an objective function

$$G(\mathbf{Q}) = \alpha \cdot \Delta\tau(\mathbf{Q}) + (1-\alpha) \cdot \Delta S(\mathbf{Q}) \qquad \text{[S-16]}$$

where $\alpha \in [0, 1]$ represents the (suggestive) weights assigned to each criteria. The Pareto frontier can be traced by solving the following constrained optimization problem for each value of α:

$$\begin{cases} \max_{\mathbf{Q}} & \alpha\Delta\tau(\mathbf{Q}) + (1-\alpha)\Delta S(\mathbf{Q}) \\ \text{s.t.} & \sum_d Q_d = \sum_d Q_d^{\mathsf{UN}} \\ & Q_d \leq R_d \end{cases} \qquad \text{[S-17]}$$

The constraint comes from the fact that the total number of resettled refugees is kept constant and equal to the UN plan. Plugging in the expressions for $\Delta\tau(\mathbf{Q})$ and $\Delta S(\mathbf{Q})$ and solving the integral, we get

$$\begin{cases} \max_{\mathbf{Q}} & \alpha \sum_d \int_0^{Q_d} -\frac{1}{\lambda_d} \ln \frac{x}{R_d} dx + (1-\alpha) \sum_d Q_d \Delta s_{d,\mathsf{SR}} \\ \text{s.t.} & \sum_d Q_d - \sum_d Q_d^{\mathsf{UN}} = 0 \\ & R_d - Q_d \geq 0 \end{cases} \qquad \text{[S-18]}$$

We solve the constrained optimization using Lagrange multipliers. Let μ and ν_d be the Lagrange multipliers for the first and second constraints, so that the Lagrangian is expressed as:

$$L = \left[\alpha \sum_d \int_0^{Q_d} -\frac{1}{\lambda_d} \ln \frac{x}{R_d} dx + (1-\alpha) \sum_d Q_d \Delta s_{d,\mathsf{SR}}\right] + \mu \left[\sum_d Q_d - \sum_d Q_d^{\mathsf{UN}}\right] + \sum_d \nu_d (R_d - Q_d) \qquad \text{[S-19]}$$

First order conditions are then:

$$\begin{cases} \frac{\partial L}{\partial Q_d} = -\frac{\alpha}{\lambda_d} \ln \frac{Q_d}{R_d} + (1-\alpha)\Delta s_{d,\mathsf{SR}} + \mu - \nu_d = 0 \forall c \\ \frac{\partial L}{\partial \mu} = \sum_d Q_d - \sum_d Q_d^{\mathsf{UN}} = 0 \forall c \\ \nu_d \frac{\partial L}{\partial \nu_d} = \nu_d \cdot (R_d - Q_d) = 0 \forall c \end{cases} \qquad \text{[S-20]}$$

The last row expresses the complementary slackness condition associated with the non binding constraint (inequality). From the first condition, we get:

$$Q_d = R_d e^{\frac{\lambda_d}{\alpha}\left((1-\alpha)\Delta s_{d,\mathsf{SR}} + \mu - \nu_d\right)} \qquad \text{[S-21]}$$

where $\Delta S_d = \sum_p \Delta S_{cp}$. Combined with the complementary slackness condition, $\nu_d \cdot (R_d - Q_d) = 0 \forall c$, we get that for each country, *either*

$$Q_d = R_d e^{\frac{\lambda_d}{\alpha}\left((1-\alpha)\Delta s_{d,\mathsf{SR}} + \mu\right)} \leq R_d \qquad \text{[S-22]}$$

or

$$Q_d = R_d. \qquad \text{[S-23]}$$

We can write this more compact as

$$Q_d = R_d \cdot \min\left\{e^{\frac{\lambda_d}{\alpha}\left((1-\alpha)\Delta s_{d,\mathsf{SR}} + \mu\right)}, 1\right\}. \qquad \text{[S-24]}$$

Plugging this into the second condition, we finally get:

$$\sum_d \left(R_d \cdot \min\left\{e^{\frac{\lambda_d}{\alpha}\left((1-\alpha)\Delta s_{d,\mathsf{SR}} + \mu\right)}, 1\right\}\right) - \sum_d Q_d^{\mathsf{UN}} = 0 \qquad \text{[S-25]}$$

For each considered value of $\alpha \in [0, 1]$, this last equation can be solved numerically to obtain μ, which can then be plugged into the previous equation for each country to obtain Q_d, and ultimately the optimal resettlement plan $\mathbf{Q}$. The plan can then itself be used to determine its effect on total individual burden of refugees $\Delta\tau(\mathbf{Q})$ and on total water stress $\Delta S(\mathbf{Q})$. To address numerical issue that emerge from the large variance in $\Delta s_{d,\mathsf{SR}}$ (>8 orders of magnitude), we carry out the transform $\beta = \frac{1-\alpha}{\alpha} \in (0, \infty)$ and solve for μ and $\mathbf{Q}$ for equally spaced values of $\log_{10} \beta$.

Robustness Check: relationship between water use and water availability The above derivation relies on the key assumption that $\Delta s_{d,\mathsf{SR}}$ is constant, meaning that the effect that a marginal resettled refugee has in terms of relieving water stress in their former country of refuge does not depend of how many refugees are being settled (Q_d). This assumption will break down if the per capita water footprint depends on per capita water availability or, in other words, if the people remaining in the country will consume more water and use up the water that is 'released' by the resettled refugee (in which case that water will not relieve the country's water stress). As a robustness check, we seek to incorporate this effect in an updated optimization. Let us assume a linear relationship between ω_{odd}, the per capita local water footprint of refugees in their current country of refuge d, and the per capita water availability in that country:

$$\omega_{odd} = \beta_0 \frac{TWA_d}{\mathsf{Pop}_d} + \beta_1 \quad \text{[S-26]}$$

where $TWA_d = (TRWR_d - EFR_d) \cdot \epsilon$ represents water availability in country d. From the above equation, we can express per capita local water footprint as a function of the number of refugees resettled:

$$\omega_{odd}(Q_d) = \beta_0 \frac{TWA_d}{\mathsf{Pop}_d - Q_d} + \beta_1$$

We now express $\Delta S(Q_d)$, the water stress relieved by Q_d resettled refugees as the difference between the 'current' water stress with a population Pop_d and the 'new' water stress with a population $\mathsf{Pop}_d - Q_d$

$$\Delta S(Q_d) = \frac{\omega_{odd} \cdot \mathsf{Pop}_d}{TWA_d} - \frac{\omega_{odd}(Q_d) \cdot (\mathsf{Pop}_d - Q_d)}{TWA_d} \quad \text{[S-27]}$$

$$= \frac{\omega_{odd} \cdot \mathsf{Pop}_d}{TWA_d} - \frac{\left(\beta_0 \frac{TWA_d}{\mathsf{Pop}_d - Q_d} + \beta_1\right) \cdot (\mathsf{Pop}_d - Q_d)}{TWA_d} \quad \text{[S-28]}$$

$$= \frac{\omega_{odd}\mathsf{Pop}_d}{TWA_d} - \beta_0 - \frac{\mathsf{Pop}_d - Q_d}{TWA_d}\beta_1 \quad \text{[S-29]}$$

[S-30]

Now consider that $\beta_1 = \omega_{odd} - \beta_0 \frac{TWA_d}{\mathsf{Pop}_d}$ and plug this into the last term of the above Equation:

$$\Delta S(Q_d) = \frac{\omega_{odd} \cdot \mathsf{Pop}_d}{TWA_d} - \beta_0 - \frac{\omega_{odd} \cdot \mathsf{Pop}_d}{TWA_d} \left(\omega_{odd} - \beta_0 \frac{TWA_d}{\mathsf{Pop}_d}\right) \quad \text{[S-31]}$$

$$= \frac{\omega_{odd} \cdot \mathsf{Pop}_d}{TWA_d} - \beta_0 - + \frac{Q_d \omega_{odd}}{TWA_d} + \beta_0 - \frac{Q_d}{\mathsf{Pop}_d}\beta_0 \quad \text{[S-32]}$$

$$= Q_d \left(\frac{\omega_{odd}}{TWA_d} - \frac{\beta_0}{\mathsf{Pop}}\right) \quad \text{[S-33]}$$

$$= Q_d \left(\Delta s_{d,\mathsf{SR}} - \frac{\beta_0}{\mathsf{Pop}}\right) \quad \text{[S-34]}$$

where $\Delta s_{d,\mathsf{SR}} = \frac{\omega_{odd}}{TWA_d}$ is the (average) marginal contribution of refugees to water stress considered in the original derivation. In words, to account for a linear relationship between per capita water consumption and availability, one simply needs to replace the marginal contribution of refugees to water stress ($\Delta s_{d,\mathsf{SR}}$) with $\left(\Delta s_{d,\mathsf{SR}} - \frac{\beta_0}{\mathsf{Pop}}\right)$, which accounts for the proportionality factor β_0 between water availability and water use. To estimate β_0 empirically, we regress annual estimates of per capita local blue water footprint (ω_{ddd}) against per capita blue water availability $\frac{TWA_d}{\mathsf{Pop}_d}$ independently for each country (see Figures S8 A for Jordan and Lebanon). Doing so yields a different value of β_0 for each country. Non-positive estimates of β_0 might arise because of structural changes of the water sector over time. For example, an increasing water efficiency might simultaneously cause water availability to increase and per capita water use to decrease. These temporal changes are assumed to be distinct from the marginal (instantaneous) effect of water availability on consumption depicted in Eqn S26. As a consequence, we set any non-positive estimate of β_0 to zero in the analysis, thus implicitly assuming that per capita water consumption is not affected by water availability in these particular countries. Countries with positive estimates of β_0 are listed in Table S8. Results of the revised analysis are displayed in Figure S8, panel B and are not materially different from the results in Figure 3B.

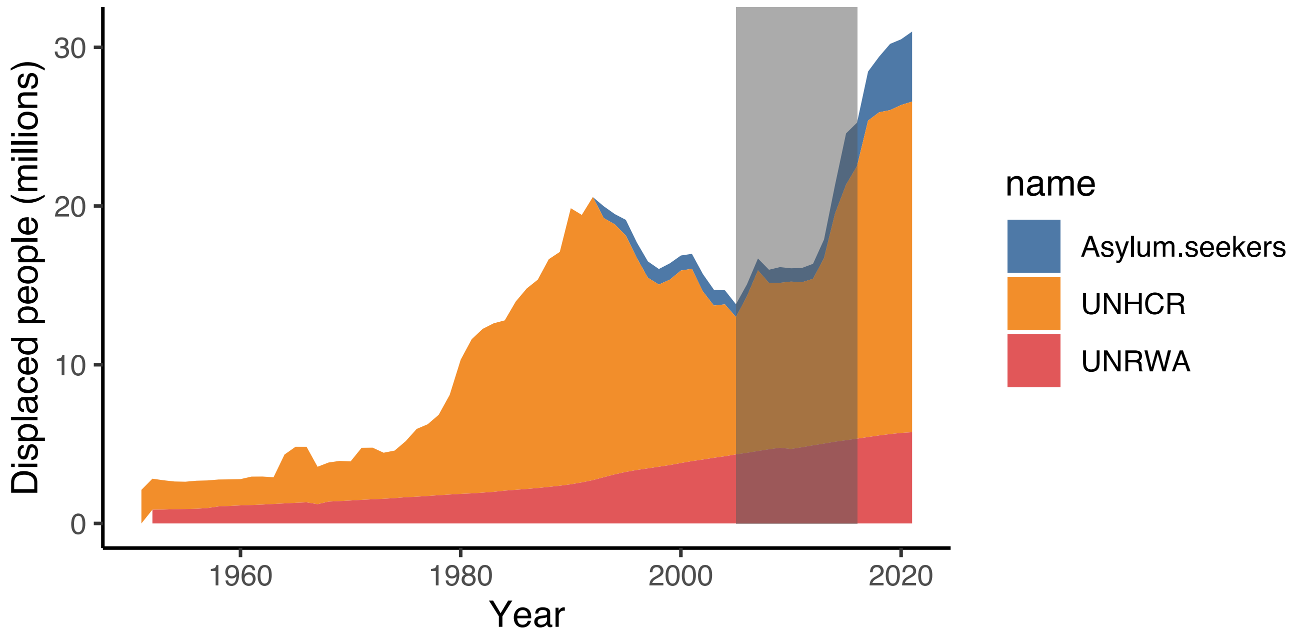

Fig. S1. Internationally displaced refugees under UNHCR and UNRWA mandates and asylum seekers between 1951 and 2020. Data from https://www.unhcr.org/refugee-statistics/

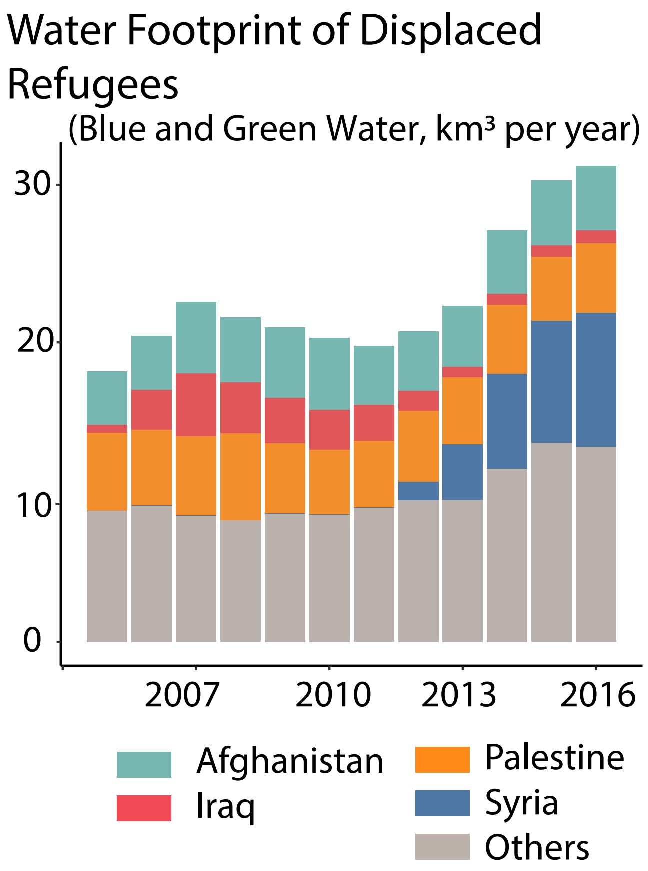

Fig. S2. Total water footprint associated with displaced refugees by country of origin. The water footprint is computed as the blue and green water demand associated with the increased food consumption in refugees' countries of destination. It excludes grey water.

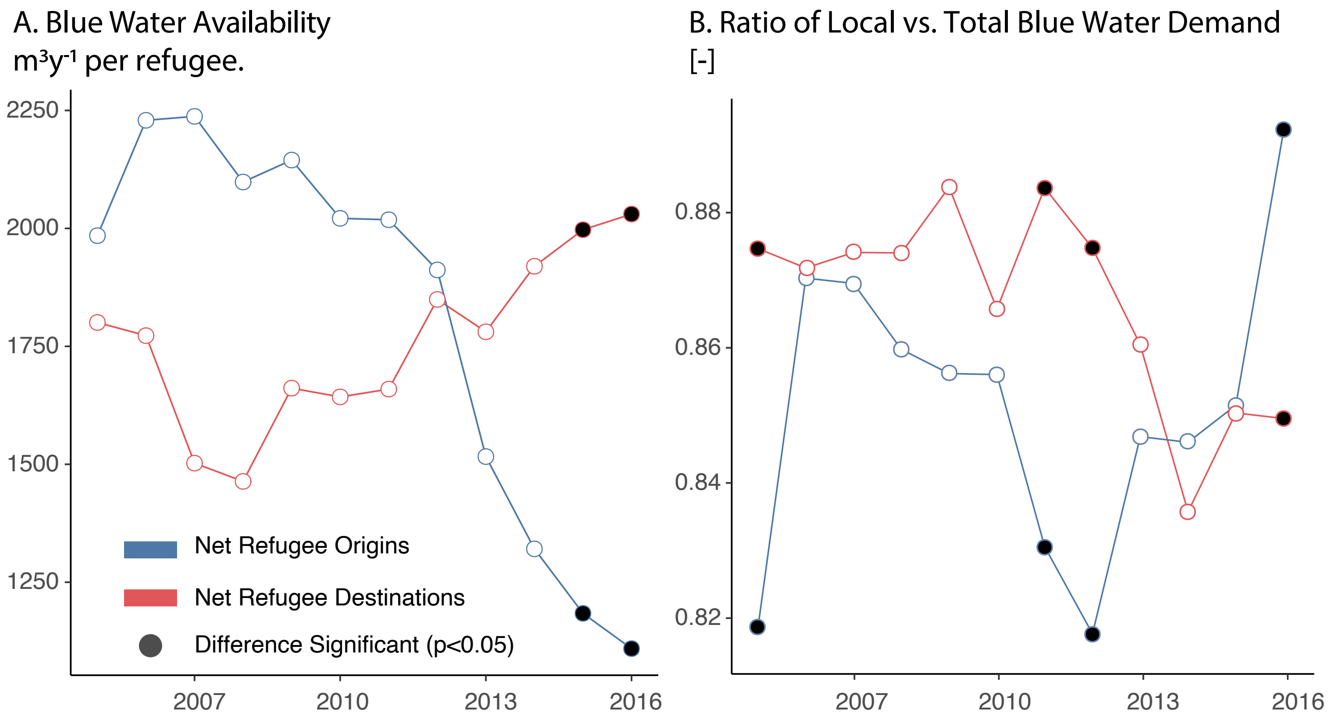

Fig. S3. A. Per capita blue water availability and **B** fraction of blue water demand satisfied using domestic water resources in net origin (blue) and destination (red) countries for refugees. Symbol represent weighted averages with net total displaced BWD as weights. Black symbols represents significant differences (one-sided t-test, $p < 0.05$) between origin and destination countries.

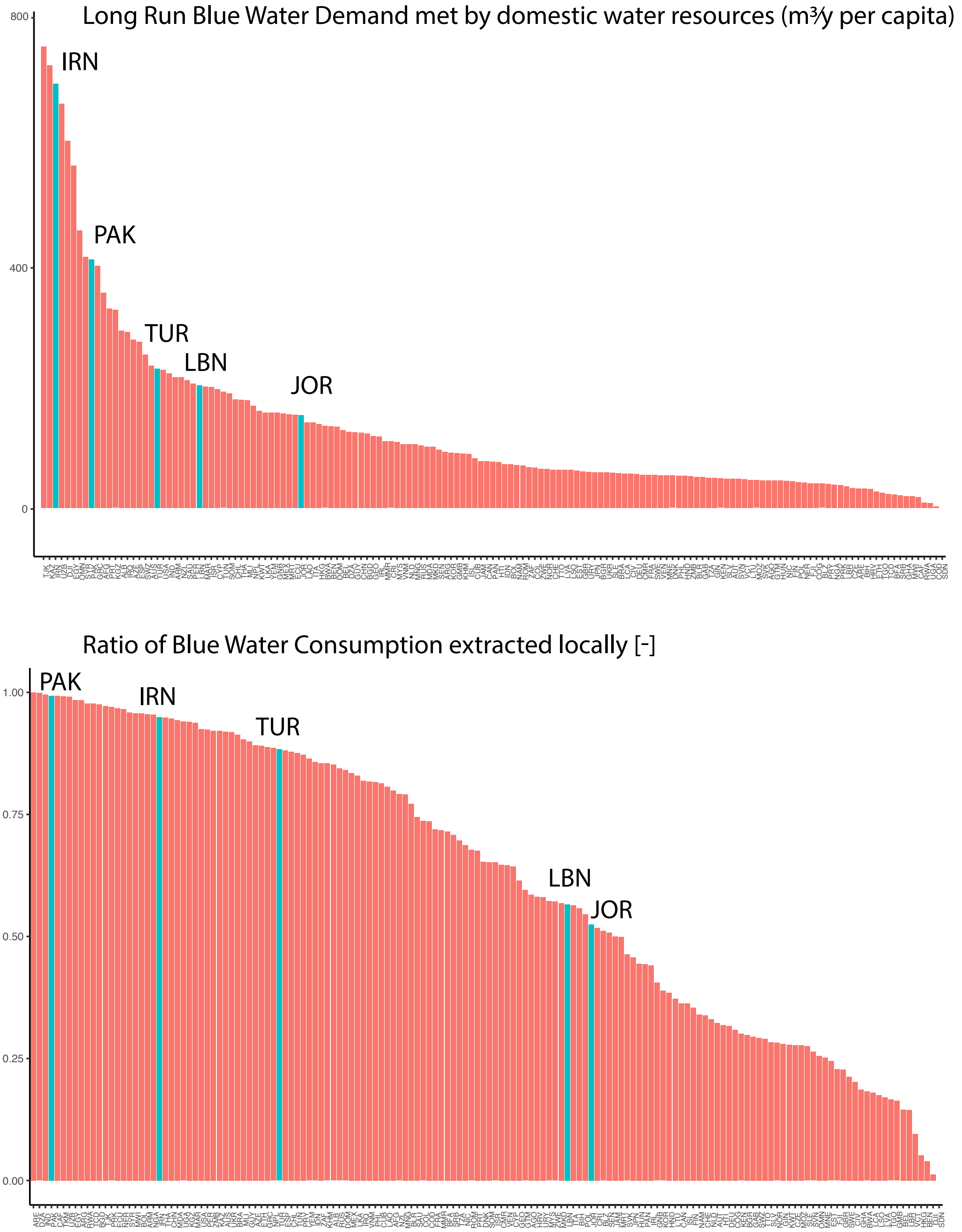

Fig. S4. A. Long run blue water demand met by domestically extracted water resources by country. Data from the CWASI dataset (1). B. Fraction of long run blue water demand met by domestically extracted water.

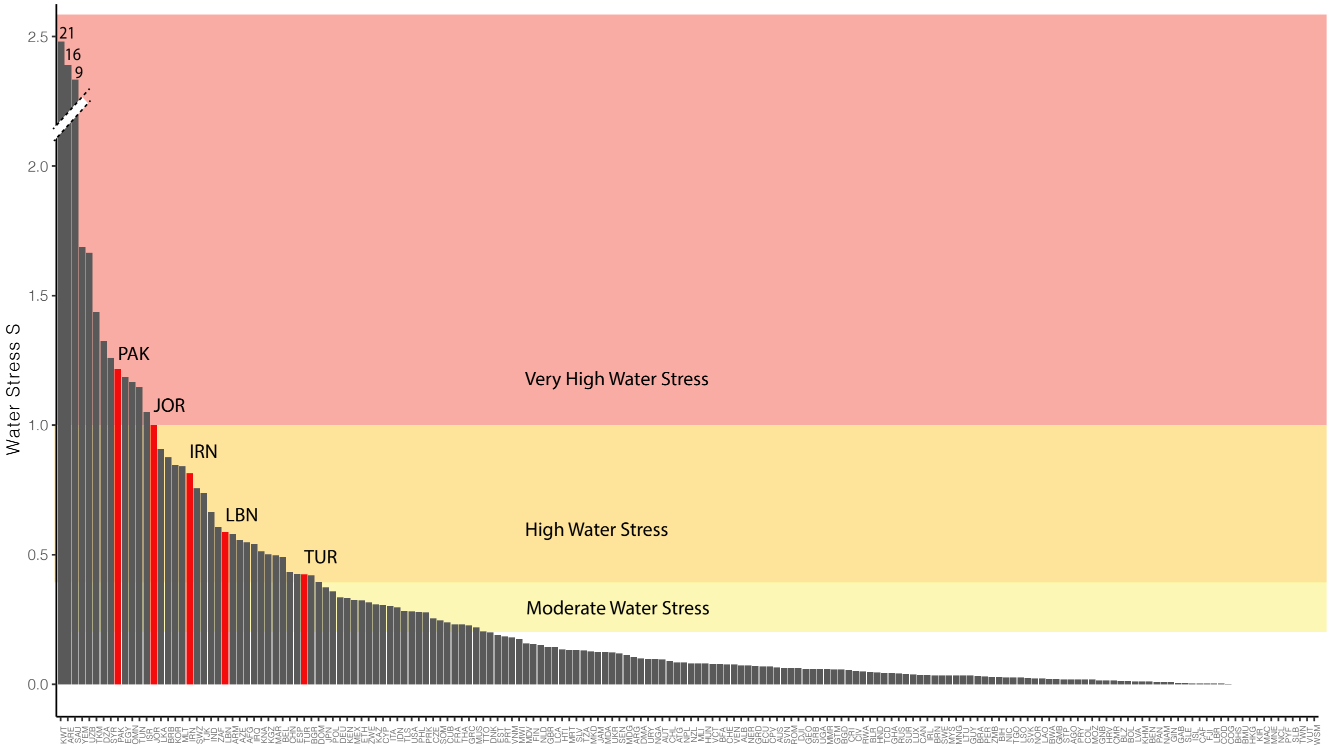

Fig. S5. Water stress index by country with thresholds for moderate ($S > 20\%$), high ($S > 40\%$) and very high ($S > 100\%$) water stress (6). Data from the Aquastat database of the Food and Agricultural Organization https://www.fao.org/aquastat/en/

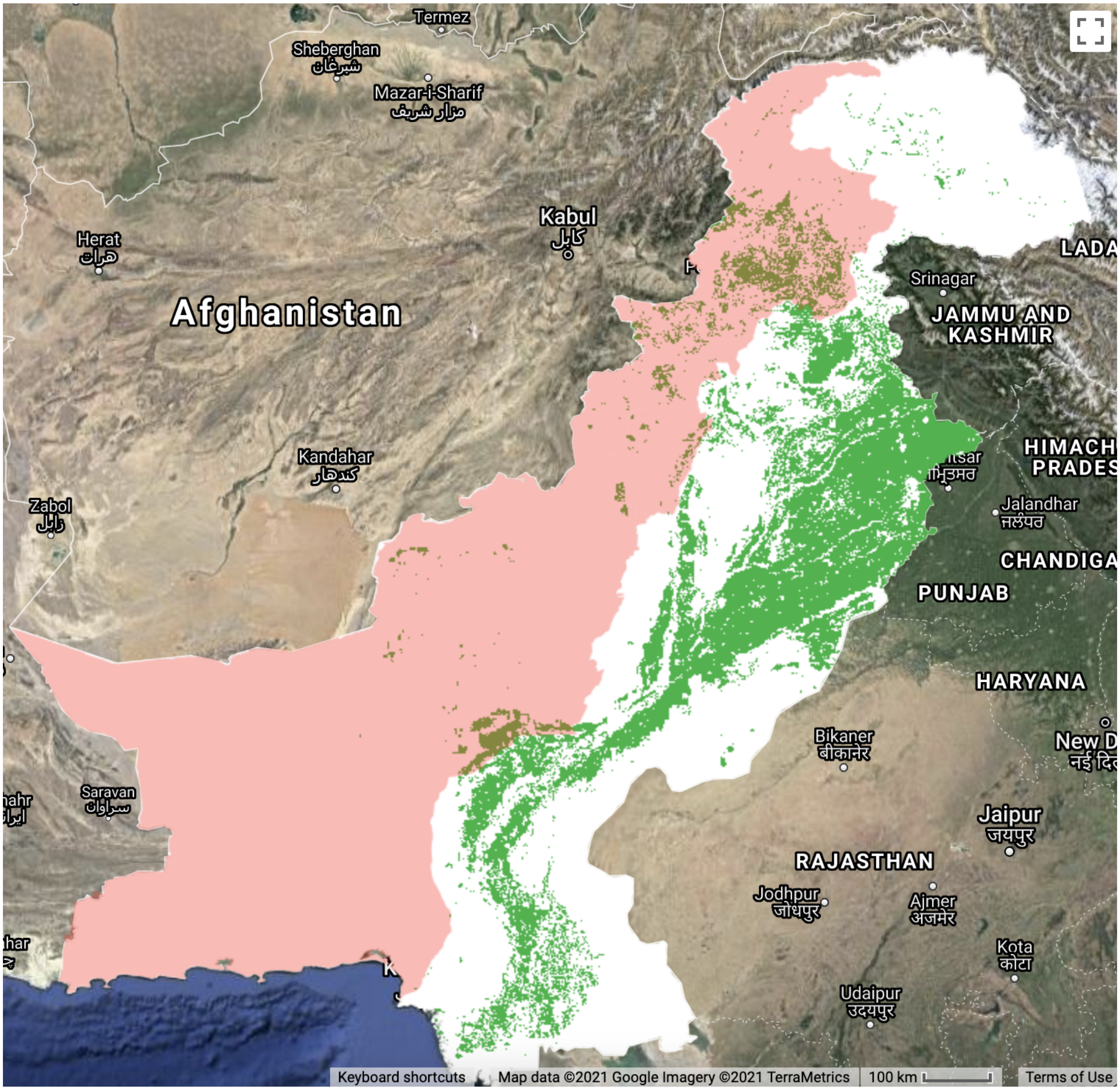

Fig. S6. Irrigated cropland in Pakistan (green) according to Global Cropland Area Database (GCAD) (7). Provinces of Pakistan that border Afghanistan are highlighted in red. Static Google Earth Engine Code available at: https://code.earthengine.google.com/ddc0ec48731daf6c057a51b210532372

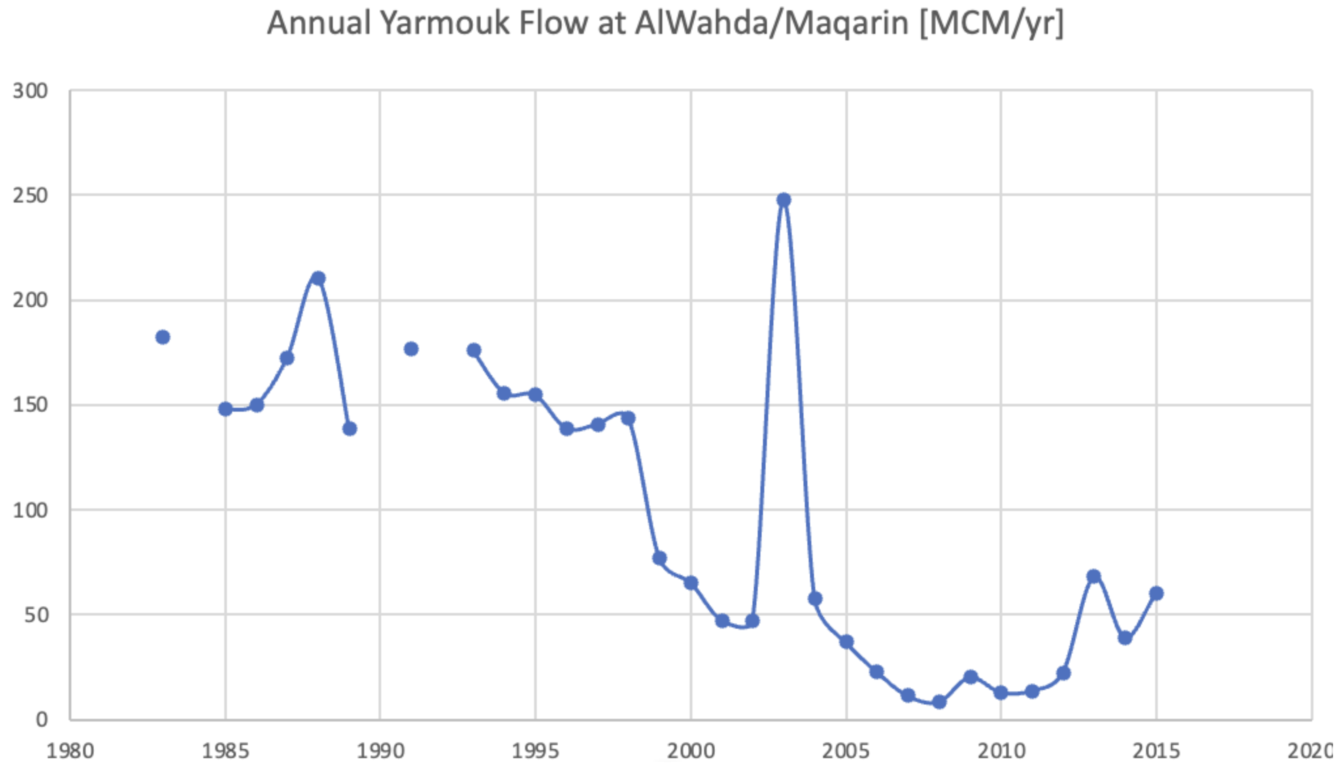

Fig. S7. Annual flow volume of the Yarmouk at Maqarin/Al Wehda between 1983 and 2015 (4)

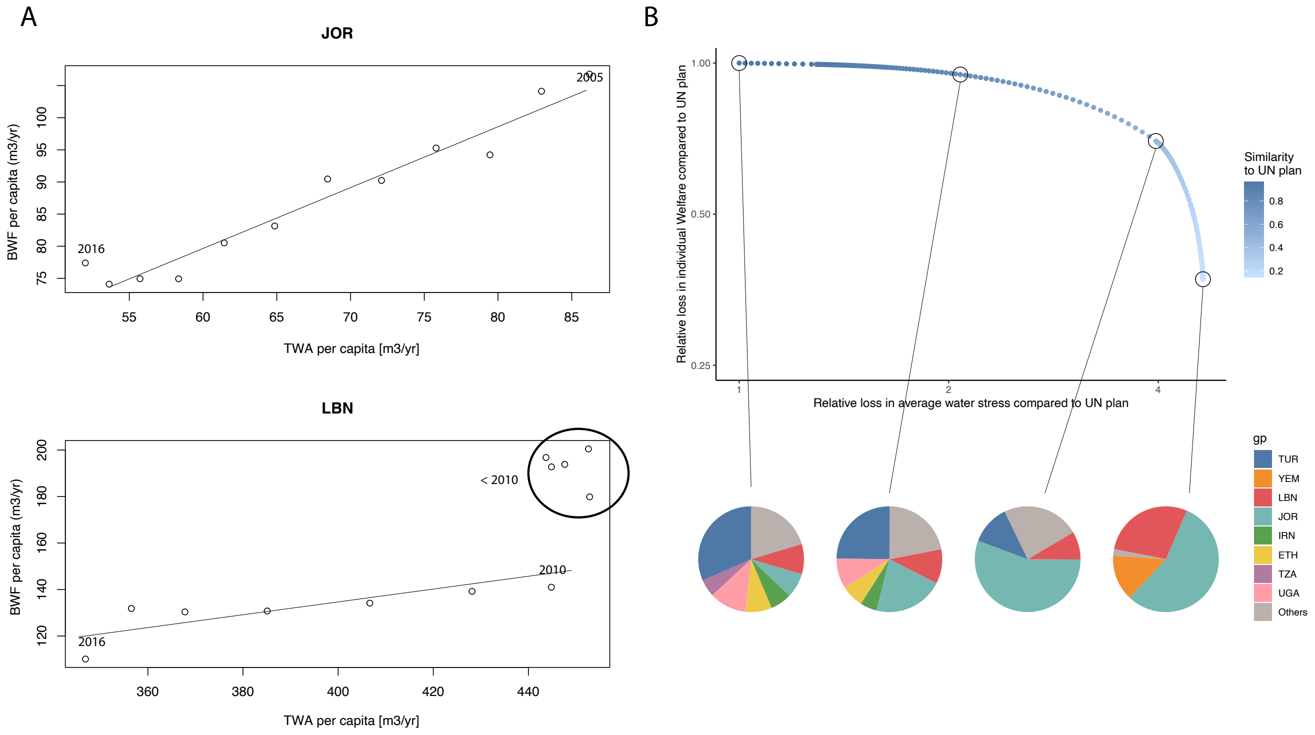

Fig. S8. Robustness check on the resettlement optimization analysis assuming a linear relationship between ω_{odd} and total water availability per capita. **Panel A**. Scatterplot of ω_{odd} vs total water availability per capita across years for Jordan and Lebanon. Both countries display an increasing relationship between the two variables that is close to linear. Observations taken prior to 2010 are clearly outliers in Lebanon and have been removed from the analysis. Per capita water use (ω_{odd}) is assumed independant from per capita water availability for countries for which the empirical relationship has a non-positive slope. **Panel B**. Outcomes of the analysis in terms of the Pareto frontier and the current countries of refuge at different points of the frontier are very similar to the original analysis in Figure 3B, which assumes no relationship between ω_{odd} and per capita water availability.

Table S1. List of countries producing or hosting at least 1000 refugees during the 2005-2016 period, which are included in the analysis. Countries with no water footprint or IWR data were addressed as described in the Methods section of the main document.

Code	Max Ref. Out	Max Ref. In	Country	Comment
ARE	590.00	7186.00	United Arab Emirates	No EFR data: EFR set to 0
DJI	2264.00	205758.00	Djibouti	No EFR data: EFR set to 0
JAM	3132.00	158.00	Jamaica	No EFR data: EFR set to 0
KWT	1665.00	98387.00	Kuwait	No EFR data: EFR set to 0
LCA	1187.00	20.00	St. Lucia	No EFR data: EFR set to 0
MLT	30.00	80206.00	Malta	No EFR data: EFR set to 0
OMN	68.00	2235.00	Oman	No EFR data: EFR set to 0
SAU	1927.00	968070.00	Saudi Arabia	No EFR data: EFR set to 0
VCT	2031.00	0.00	St. Vincent & Grenadines	No EFR data: EFR set to 0
YEM	34298.00	2339194.00	Yemen	No EFR data: EFR set to 0
NA	1167442.00	0.00	Unknown	No Footprint or IWR
BDI	446955.00	510512.00	Burundi	No Footprint or IWR
BHR	578.00	2674.00	Bahrain	No Footprint or IWR
BIH	201633.00	91396.00	Bosnia & Herzegovina	No Footprint or IWR
BLZ	218.00	5815.00	Belize	No Footprint or IWR
BTN	112231.00	0.00	Bhutan	No Footprint or IWR
CAF	501479.00	191120.00	Central African Republic	No Footprint or IWR
CHL	1478.00	27057.00	Chile	No Footprint or IWR
CRI	925.00	198914.00	Costa Rica	No Footprint or IWR
ERI	524932.00	53816.00	Eritrea	No Footprint or IWR
ESH	118643.00	0.00	Western Sahara	No Footprint or IWR
FJI	2246.00	66.00	Fiji	No Footprint or IWR
HKG	125.00	20359.00	Hong Kong SAR China	No Footprint or IWR
HND	45707.00	357.00	Honduras	No Footprint or IWR
HRV	119364.00	16121.00	Croatia	No Footprint or IWR
ISL	32.00	2626.00	Iceland	No Footprint or IWR
LBR	237107.00	421653.00	Liberia	No Footprint or IWR
LBY	15348.00	229854.00	Libya	No Footprint or IWR
MNE	4422.00	122798.00	Montenegro	No Footprint or IWR
NRU	5.00	3656.00	Nauru	No Footprint or IWR
PAN	145.00	189094.00	Panama	No Footprint or IWR
PNG	662.00	117697.00	Papua New Guinea	No Footprint or IWR
PSE	3285937.00	0.00	Palestinian Territories	No Footprint or IWR
QAT	128.00	1546.00	Qatar	No Footprint or IWR
SDN	707085.00	2670947.00	Sudan	No Footprint or IWR
SLV	81782.00	563.00	El Salvador	No Footprint or IWR
SSD	1442504.00	1313832.00	South Sudan	No Footprint or IWR
TIB	20208.00	0.00	Tibet	No Footprint or IWR
XXA	59434.00	0.00	Sateless	No Footprint or IWR
LIE	0.00	2151.00	Liechtenstein	No Footprint or IWR
LUX	0.00	38341.00	Luxembourg	No Footprint or IWR
SRB	208117.00	913708.00	Serbia	Population data interpolated for 2008
AFG	3091800.00	660910.00	Afghanistan	
AGO	224148.00	342629.00	Angola	
ALB	52561.00	5480.00	Albania	
ARG	1282.00	53691.00	Argentina	
ARM	23800.00	424091.00	Armenia	
AUS	57.00	538212.00	Australia	
AUT	62.00	1041732.00	Austria	
AZE	237797.00	23584.00	Azerbaijan	
BEL	115.00	492979.00	Belgium	
BEN	1624.00	89076.00	Benin	
BFA	6549.00	177128.00	Burkina Faso	
BGD	42701.00	1993546.00	Bangladesh	
BGR	5459.00	132970.00	Bulgaria	
BLR	11060.00	11165.00	Belarus	
BOL	940.00	8526.00	Bolivia	

Continued on next page

Table S1 – continued from previous page

Code	Max Ref. Out	Max Ref. In	Country	Comment
BRA	4562.00	160842.00	Brazil	
BWA	390.00	35185.00	Botswana	
CAN	545.00	2259426.00	Canada	
CHE	38.00	885081.00	Switzerland	
CHN	279929.00	3631310.00	China	
CIV	173011.00	216194.00	CÃŽte dâIvoire	
CMR	21283.00	1783114.00	Cameroon	
COD	620179.00	2393183.00	Congo - Kinshasa	
COG	32600.00	915975.00	Congo - Brazzaville	
COL	594839.00	3673.00	Colombia	
CUB	34901.00	5485.00	Cuba	
CYP	15.00	114033.00	Cyprus	
CZE	3793.00	44411.00	Czechia	
DEU	258.00	8006595.00	Germany	
DNK	26.00	311501.00	Denmark	
DOM	2716.00	11889.00	Dominican Republic	
DZA	13405.00	1155349.00	Algeria	
ECU	14721.00	1607677.00	Ecuador	
EGY	32816.00	1916117.00	Egypt	
ESP	2457.00	121441.00	Spain	
EST	870.00	1013.00	Estonia	
ETH	162809.00	3946193.00	Ethiopia	
FIN	11.00	173462.00	Finland	
FRA	342.00	3057402.00	France	
GAB	597.00	99354.00	Gabon	
GBR	237.00	2795608.00	United Kingdom	
GEO	20549.00	17331.00	Georgia	
GHA	33670.00	311395.00	Ghana	
GIN	41927.00	245378.00	Guinea	
GMB	30472.00	126983.00	Gambia	
GNB	3512.00	99768.00	Guinea-Bissau	
GRC	375.00	521529.00	Greece	
GTM	58772.00	2921.00	Guatemala	
GUY	1048.00	69.00	Guyana	
HTI	52126.00	56.00	Haiti	
HUN	7237.00	131526.00	Hungary	
IDN	38880.00	68792.00	Indonesia	
IND	35226.00	2219952.00	India	
IRL	88.00	143498.00	Ireland	
IRN	181636.00	11591457.00	Iran	
IRQ	2336917.00	1495216.00	Iraq	
ISR	2523.00	373119.00	Israel	
ITA	240.00	1059402.00	Italy	
JOR	4349.00	29923441.00	Jordan	
JPN	538.00	96350.00	Japan	
KAZ	8027.00	34097.00	Kazakhstan	
KEN	16068.00	5438194.00	Kenya	
KGZ	4931.00	24786.00	Kyrgyzstan	
KHM	18662.00	2061.00	Cambodia	SR per capita BWD of KHM in VNM unavailable
KOR	1641.00	32592.00	South Korea	
LAO	26728.00	0.00	Laos	
LBN	20321.00	9668129.00	Lebanon	
LKA	153272.00	9001.00	Sri Lanka	
LSO	1107.00	225.00	Lesotho	
LTU	1622.00	11001.00	Lithuania	
LVA	2545.00	2521.00	Latvia	
MAR	9490.00	33066.00	Morocco	
MDA	12950.00	3343.00	Moldova	

Continued on next page

Table S1 – continued from previous page

Code	Max Ref. Out	Max Ref. In	Country	Comment
MEX	74647.00	37302.00	Mexico	
MKD	16380.00	18728.00	North Macedonia	
MLI	166113.00	175367.00	Mali	
MMR	546305.00	0.00	Myanmar (Burma)	
MNG	6113.00	118.00	Mongolia	
MOZ	5746.00	142313.00	Mozambique	
MRT	46367.00	543346.00	Mauritania	
MWI	8305.00	188980.00	Malawi	
MYS	6615.00	1139129.00	Malaysia	
NAM	2158.00	68076.00	Namibia	
NER	2144.00	479681.00	Niger	
NGA	296157.00	82865.00	Nigeria	
NIC	5886.00	2819.00	Nicaragua	
NLD	196.00	1166396.00	Netherlands	
NOR	25.00	645279.00	Norway	
NPL	19889.00	988660.00	Nepal	
NZL	43.00	32641.00	New Zealand	
PAK	380359.00	19007081.00	Pakistan	
PER	12075.00	22759.00	Peru	
PHL	3540.00	2853.00	Philippines	
POL	20246.00	189870.00	Poland	
PRK	2153.00	0.00	North Korea	
PRT	196.00	8784.00	Portugal	
PRY	185.00	1414.00	Paraguay	
ROU	12630.00	24043.00	Romania	
RUS	178443.00	827242.00	Russia	
RWA	297754.00	886901.00	Rwanda	
SEN	39358.00	261817.00	Senegal	
SLE	48474.00	140830.00	Sierra Leone	
SOM	1179814.00	161451.00	Somalia	
SVK	1372.00	14314.00	Slovakia	
SVN	1767.00	4409.00	Slovenia	
SWE	85.00	1794094.00	Sweden	
SWZ	373.00	11937.00	Eswatini	
SYR	5708842.00	12862944.00	Syria	
TCD	58429.00	4268996.00	Chad	
TGO	58556.00	173681.00	Togo	
THA	3524.00	1496539.00	Thailand	
TJK	54904.00	35911.00	Tajikistan	
TKM	1650.00	13300.00	Turkmenistan	
TTO	564.00	1321.00	Trinidad & Tobago	
TUN	4202.00	10783.00	Tunisia	
TUR	239421.00	8620828.00	Turkey	
TZA	6777.00	2952402.00	Tanzania	
UGA	38510.00	3788684.00	Uganda	
UKR	343754.00	91395.00	Ukraine	
URY	245.00	2943.00	Uruguay	
USA	5145.00	5472875.00	United States	
UZB	14169.00	49473.00	Uzbekistan	
VEN	52615.00	2021643.00	Venezuela	
VNM	376036.00	14703.00	Vietnam	
ZAF	1530.00	4380748.00	South Africa	
ZMB	762.00	766389.00	Zambia	
ZWE	78705.00	75040.00	Zimbabwe	

Table S2. Short run (SR) and long run (LR) blue Water demand and water stress associated with refugee displacement by destination countries. For each destination country, values are given for the year with maximum short run increase in water stress during the 2005-2016 period, indicated in the Year column.

Dest.	Year	Pop. 10^6	Stress %	Refug. 10^6	Δ BWD MCM/y		Δ Stress %-points	
					SR	LR	SR	LR
JOR	2016	9.6	100	3.3	395	260	79.4	52.2
KWT	2007	2.5	2075	0.0	1	2	22.3	59.7
LBN	2016	6.7	59	1.7	288	184	12.4	7.9
ARE	2010	8.5	1581	0.0	12	6	9.7	5.3
SYR	2007	19.9	126	1.9	520	659	9.5	12.1
SAU	2006	24.5	943	0.2	91	90	6.8	6.8
YEM	2013	25.1	169	0.2	35	38	3.1	3.3
ARM	2005	3.0	49	0.2	33	35	1.6	1.7
ISR	2016	8.1	105	0.1	6	8	1.1	1.5
ZAF	2015	55.4	60	1.2	63	75	1.1	1.3
CYP	2016	1.2	31	0.0	3	2	0.8	0.5
SWZ	2010	1.1	76	0.0	3	2	0.6	0.5
IRN	2009	72.9	81	1.0	360	675	0.6	1.0
PAK	2009	175.5	114	1.7	601	728	0.5	0.6
EGY	2006	76.9	114	0.1	205	156	0.5	0.4
OMN	2005	2.5	91	0.0	3	6	0.4	0.9
KAZ	2014	17.3	30	0.0	160	0	0.4	0.0
ETH	2016	103.6	32	0.6	19	15	0.4	0.3
DJI	2011	0.9	6	0.0	0	0	0.3	0.2
TUN	2016	11.3	115	0.0	6	2	0.3	0.1
TUR	2016	79.8	42	3.0	227	568	0.3	0.7
KEN	2011	43.2	28	0.6	11	9	0.3	0.2
DZA	2010	36.0	104	0.1	13	13	0.2	0.2
GHA	2014	27.2	4	0.0	9	0	0.2	0.0
NER	2016	20.8	7	0.2	9	7	0.2	0.2
IDN	2014	255.1	28	0.0	455	-26	0.2	-0.0
GEO	2016	4.0	6	0.0	20	-1	0.2	-0.0
UGA	2016	39.6	6	1.0	5	7	0.2	0.3
BFA	2014	17.6	8	0.0	5	0	0.2	0.0
ZMB	2015	15.9	3	0.0	21	2	0.1	0.0
MWI	2008	13.7	18	0.0	3	1	0.1	0.0
IND	2014	1295.6	66	0.2	633	67	0.1	0.0
DNK	2016	5.7	20	0.0	1	3	0.1	0.3
MYS	2014	29.9	3	0.1	108	3	0.1	0.0
ESP	2016	46.6	43	0.0	55	39	0.1	0.1
IRQ	2015	35.6	57	0.2	27	-50	0.1	-0.2
ECU	2009	14.8	7	0.2	50	29	0.1	0.1
TKM	2005	4.8	144	0.0	9	21	0.1	0.2
MRT	2012	3.7	13	0.0	3	3	0.1	0.1
ITA	2010	59.3	42	0.1	49	12	0.1	0.0
MAR	2016	35.1	50	0.0	8	10	0.1	0.1
UZB	2005	26.4	141	0.0	8	26	0.1	0.2
CZE	2016	10.6	25	0.0	0	0	0.1	0.0
TGO	2014	7.1	3	0.0	0	0	0.1	0.0
DEU	2016	82.2	33	1.3	8	25	0.0	0.1
TZA	2005	38.5	13	0.5	3	18	0.0	0.2
GRC	2016	10.6	23	0.1	13	35	0.0	0.1
CMR	2016	23.9	1	0.4	6	12	0.0	0.1
TJK	2011	7.7	74	0.0	2	3	0.0	0.0
BEL	2016	11.4	49	0.1	1	3	0.0	0.1
PRT	2014	10.4	18	0.0	8	1	0.0	0.0
THA	2009	66.9	23	0.1	48	24	0.0	0.0
UKR	2012	45.5	17	0.0	7	11	0.0	0.0
NPL	2005	25.7	8	0.1	18	17	0.0	0.0

Continued on next page

Table S2 – continued from previous page

Dest.	Year	Pop. 10^6	Stress %	Refug. 10^6	Δ BWD MCM/y SR	Δ BWD MCM/y LR	Δ Stress %-points SR	Δ Stress %-points LR
FRA	2016	64.7	23	0.4	27	31	0.0	0.0
DOM	2009	9.6	38	0.0	1	-0	0.0	-0.0
AUS	2006	20.5	7	0.1	29	38	0.0	0.0
KGZ	2011	5.5	50	0.0	1	1	0.0	0.0
TCD	2016	14.6	4	0.4	1	8	0.0	0.1
GUY	2010	0.7	3	0.0	3	0	0.0	0.0
MDA	2014	4.1	13	0.0	1	2	0.0	0.0
AGO	2016	28.8	2	0.0	2	1	0.0	0.0
POL	2016	38.0	36	0.0	2	3	0.0	0.0
HUN	2015	9.8	8	0.0	1	1	0.0	0.0
URY	2005	3.3	10	0.0	2	3	0.0	0.0
SVK	2006	5.4	3	0.0	1	0	0.0	0.0
ZWE	2009	12.5	33	0.0	1	0	0.0	0.0
COD	2015	76.2	0	0.2	13	-0	0.0	-0.0
USA	2016	323.0	28	0.7	153	205	0.0	0.0
VEN	2009	28.0	8	0.2	17	12	0.0	0.0
NZL	2016	4.7	8	0.0	9	3	0.0	0.0
MLI	2010	15.0	8	0.0	2	2	0.0	0.0
BGR	2015	7.2	42	0.0	1	1	0.0	0.0
IRL	2016	4.7	4	0.0	1	2	0.0	0.0
CHE	2016	8.4	8	0.1	1	2	0.0	0.0
GMB	2012	1.9	2	0.0	0	0	0.0	0.0
GTM	2015	16.3	6	0.0	1	-0	0.0	-0.0
NLD	2016	17.0	15	0.1	5	8	0.0	0.0
GBR	2016	66.3	14	0.2	2	3	0.0	0.0
JAM	2006	2.8	8	0.0	0	0	0.0	0.0
SEN	2008	12.0	12	0.0	1	1	0.0	0.0
BGD	2014	154.5	6	0.2	30	23	0.0	0.0
CUB	2016	11.3	24	0.0	0	5	0.0	0.1
ROM	2016	19.8	6	0.0	5	14	0.0	0.0
BEN	2005	8.0	1	0.0	0	0	0.0	0.0
MEX	2014	120.4	32	0.0	6	5	0.0	0.0
BRA	2009	193.9	3	0.0	44	4	0.0	0.0
ARG	2009	40.5	10	0.0	7	4	0.0	0.0
GNB	2008	1.4	1	0.0	0	0	0.0	0.0
MOZ	2016	27.8	2	0.0	1	1	0.0	0.0
RUS	2016	145.3	4	0.1	38	60	0.0	0.0
CIV	2005	18.4	7	0.0	0	0	0.0	0.0
GIN	2005	9.1	1	0.1	0	1	0.0	0.0
KOR	2016	51.0	85	0.0	0	0	0.0	0.0
SWE	2016	9.8	3	0.3	1	4	0.0	0.0
CAN	2011	34.5	4	0.2	12	6	0.0	0.0
NAM	2009	2.1	1	0.0	0	0	0.0	0.0
CHN	2011	1376.5	43	0.1	17	13	0.0	0.0
BWA	2005	1.8	2	0.0	0	0	0.0	0.0
GAB	2010	1.6	1	0.0	0	0	0.0	0.0
PRY	2014	6.6	2	0.0	2	0	0.0	0.0
NIC	2014	6.1	3	0.0	0	0	0.0	0.0
PER	2016	30.9	3	0.0	3	2	0.0	0.0
AUT	2006	8.3	10	0.1	1	1	0.0	0.0
KHM	2015	15.5	1	0.0	1	-1	0.0	-0.0
EST	2016	1.3	19	0.0	0	0	0.0	0.0
COG	2016	5.0	0	0.0	0	0	0.0	0.0
SVN	2016	2.1	6	0.0	0	0	0.0	0.0
SRB	2016	8.9	6	0.0	0	0	0.0	0.0
JPN	2016	127.8	37	0.0	1	1	0.0	0.0

Continued on next page

Table S2 – continued from previous page

Dest.	Year	Pop. 10^6	Stress %	Refug. 10^6	Δ BWD MCM/y SR	LR	Δ Stress %-points SR	LR
NGA	2006	142.5	9	0.0	0	-0	0.0	-0.0
LTU	2014	3.0	6	0.0	0	0	0.0	0.0
BLR	2016	9.4	5	0.0	1	-0	0.0	-0.0
BOL	2009	9.9	1	0.0	0	-0	0.0	-0.0
PHL	2006	87.9	25	0.0	0	0	0.0	0.0
LVA	2016	2.0	1	0.0	0	0	0.0	0.0
LSO	2005	2.0	3	0.0	-0	-0	-0.0	-0.0
TTO	2006	1.3	19	0.0	-0	0	-0.0	0.0
SLE	2005	5.6	0	0.0	-0	0	-0.0	0.0
PRK	2005	23.9	28	0.0	-0	-0	-0.0	-0.0
MNG	2005	2.5	4	0.0	-0	-0	-0.0	-0.0
LAO	2008	6.0	2	0.0	-1	-1	-0.0	-0.0
COL	2005	42.6	2	0.1	-3	-3	-0.0	-0.0
LCA	2005	0.2	14	0.0	-0	-0	-0.0	-0.0
MMR	2005	48.9	6	0.2	-14	-14	-0.0	-0.0
RWA	2013	10.8	5	0.0	-0	-0	-0.0	-0.0
VCT	2005	0.1	9	0.0	-0	-0	-0.0	-0.0
VNM	2009	87.1	18	0.3	-23	-24	-0.0	-0.0
MKD	2016	2.1	13	0.0	-0	-0	-0.0	-0.0
HTI	2005	9.2	13	0.0	-1	-1	-0.0	-0.0
AZE	2014	9.5	54	0.0	-2	-3	-0.0	-0.0
ALB	2005	3.1	8	0.0	-3	-3	-0.0	-0.0
LKA	2005	19.5	91	0.1	-13	-13	-0.3	-0.3
SOM	2009	11.7	25	0.7	-21	-98	-1.9	-9.1
AFG	2015	34.4	55	-2.7	-500	-714	-2.5	-3.5

Table S3. The water footprint of in-kind food consumption was obtained from (8) for for the top nine recipient of virtual water embedded in food aid in 2005. The water footprint of crop production and trade were obtained from (9) for circa 2005 and used to obtain Net Consumption through mass balance, assuming market clearing. We compare food aid import to crop production alone and disregard the footprint of other agricultural product. This is conservative for our purpose as it likely overestimates the ratio of aid to consumption. All footprint values are given in Million Cubic Meters per year (MCM/y) of blue and green water.

	In Kind Food Aid MCM/y	Crop Production MCM/y	Import MCM/y	Export MCM/y	Net Consumption MCM/y	Ratio of Aid to Consumption %
ETH	1996	57985	1330	2334	56981	3.5
SDN	1405	49320	1925	5042	46204	3.0
PRK	1147	11994	2250	127	14117	8.1
BGD	448	80882	17497	8073	90307	0.5
AFG	408	15669	2316	1086	16899	2.4
ERI	375	1799	2559	37	4322	8.7
UGA	300	33840	1380	3634	31586	0.9
HTI	295	6036	1597	764	6869	4.3
KEN	241	18090	3924	3766	18248	1.3

Table S4. Correspondence between families of foods in the FAO Balance sheet in the families used in our analysis based on the CWASI dataset (New Family)

FAOBalance.Number	FAOBalance.Name	NewFamily.Number	NewFamily.Name
2511	Wheat and products	2511	Wheat
2805	Rice (Milled Equivalent)	2805	Rice
2513	Barley and products	2513	Barley
2656	Beer	2513	Barley
2514	Maize and products	2514	Maize
2582	Maize Germ Oil	2514	Maize
2515	Rye and products	2515	Rye

2516	Oats	2516	Oats
2517	Millet and products	2517	Millet
2518	Sorghum and products	2518	Sorghum
2520	Cereals, Other	2520	Cereals, Other
2531	Potatoes and products	2531	Potatoes
2533	Sweet potatoes	2533	Sweet potatoes
2532	Cassava and products	2532	Cassava
2534	Roots, Other	2534	Roots, Other
2535	Yams	2535	Yams
2536	Sugar cane	2536	Sugar
2537	Sugar beet	2536	Sugar
2543	Sweeteners, Other	2543	Sweeteners, Other
2542	Sugar (Raw Equivalent)	2536	Sugar
2541	Sugar non-centrifugal	2536	Sugar
2546	Beans	2546	Beans
2549	Pulses, Other and products	2549	Pulses, Other
2547	Peas	2547	Peas
2551	Nuts and products	2551	Nuts and proucts
2555	Soyabeans	2555	Soyabeans
2571	Soyabean Oil	2555	Soyabeans
2556	Groundnuts (Shelled Eq)	2556	Groundnuts
2572	Groundnut Oil	2556	Groundnuts
2560	Coconuts - Incl Copra	2560	Coconuts
2578	Coconut Oil	2560	Coconuts
2562	Palm kernels	2562	Palm kernels
2577	Palm oil	2562	Palm kernels
2576	Palm kernel oil	2562	Palm kernels
2563	Olives (including preserved)	2563	Olives
2580	Olive Oil	2563	Olives
2570	Oilcrops, Other	2570	Oilcrops, Other
2586	Oilcrops Oil, Other	2570	Oilcrops, Other
2557	Sunflower seed	2557	Sunflower seed
2573	Sunflowerseed Oil	2557	Sunflower seed
2558	Rape and Mustardseed	2558	Rape and Mustardseed
2574	Rap and Mustard Oil	2558	Rape and Mustardseed
2561	Sesame seed	2561	Sesame seed
2579	Sesameseed Oil	2561	Sesame seed
2575	Cottonseed Oil	2559	Cottonseed
2605	Vegetables, Other	2605	Vegetables, Other
2601	Tomatoes and products	2601	Tomatoes
2602	Onions	2602	Onions
2615	Bananas	2615	Bananas
2616	Plantains	2616	Plantains
2611	Oranges, Mandarines	2611	Oranges, Mandarines
2612	Lemons, Limes and products	2612	Lemons, Limes
2613	Grapefruit and products	2613	Grapefruit
2614	Citrus, Other	2614	Citrus, Other
2617	Apples and products	2617	Apples
2625	Fruits, Other	2625	Fruits, Other
2620	Grapes and products (exl wine)	2620	Grapes
2655	Wine	2620	Grapes
2618	Pineapples and products	2618	Pineapples
2619	Dates	2619	Dates
2658	Beverages, Alcoholic	2658	Beverages, Alcoholic
2630	Coffee and products	2630	Coffee
2633	Cocoa Beans and products	2633	Cocoa
2586	Oilcrops Oil, Other	2570	Oilcrops, Other
2635	Tea (including mate)	2635	Tea
2636	Tea (including mate)	2635	Tea
2640	Pepper	2640	Pepper
2641	Pimento	2641	Pimento

2645	Spices, Other	2645	Spices, Other
2642	Cloves	2642	Cloves
2731	Bovine Meat	2731	Bovine Meat
2736	Offals, Edible	2736	Offals, Edible
2737	Fats, Animals, Raw	2737	Fats, Animals, Raw
2848	Milk - Excluding Butter	2848	Milk - Excluding Butter
2743	Cream	2743	Cream
2740	Butter, Ghee	2740	Butter, Ghee
2732	Mutton & Goat Meat	2732	Mutton & Goat Meat
2733	Pigmeat	2733	Pigmeat
2734	Poultry Meat	2734	Poultry Meat
2744	Eggs	2744	Eggs
2735	Meat, Other	2735	Meat, Other

Table S5. Cross validation results on the regression-based estimation of Φ and D

Country	WAPE on Φ	WAPE on Z'
Central African Republic	0.46	0.48
Egypt	0.20	0.32
Ethiopia	0.33	0.39
Jordan	0.27	0.40
Kenya	0.28	0.31
Lebanon	0.32	0.41
Tanzania	0.28	0.39
Uganda	0.49	0.49
Zambia	0.31	0.40

Table S6. Reliance on virtual water import regressed against refugee immigration, 149 countries, 2005-2016

	Dependent variable (Ordinary Least Squares):	
	Ratio of long run per capita BWD satisfied through import	
	(1)	(2)
Refugees (10^6 persons)	-0.086^{***}	0.020
	(0.023)	(0.029)
Constant	0.376^{***}	0.183^{***}
	(0.008)	(0.043)
Observations	1,720	1,720
Fixed Effects	No	Country

Note: *:$p < 0.1$; **:$p < 0.05$; ***:$p < 0.01$, from two-sided t-tests that the corresponding regression coefficient is different from zero.

Table S7. Negative virtual water fluxes: $\%C$ represents the percentage of countries ($N = 131$) where negative water fluxes represent more than 5% of per capita blue water footprints; $\%R$ represents the percentage of global refugees that these countries host

Year	$\%C$	$\%R$
2005	5.5	6.0
2006	6.1	4.8
2007	3.6	4.6
2008	5.5	5.0
2009	4.2	4.7
2010	3.7	4.8
2011	4.2	4.9
2012	6.1	5.1
2013	5.4	4.8
2014	7.3	4.1
2015	6.0	4.2
2016	6.7	4.1

Table S8. Countries with positive regression coefficient β_0 between per capita local blue water footprint (ω_{odd}) and per capita water availability.

Country	β_0
AFG	0.37
ARE	17.89
AUS	0.03
BGD	0.01
BLR	0.02
BRA	0.01
BWA	0.02
CHE	0.01
CYP	0.56
CZE	0.02
DOM	0.01
DZA	0.48
EGY	0.40
ESP	0.03
ETH	0.08
GNB	0.02
GUY	0.15
HTI	0.07
HUN	0.02
IND	0.04
IRN	0.65
IRQ	2.49
ISR	1.13
ITA	0.06
JOR	0.95
KGZ	0.33
KWT	5.92
LBN	0.22
LSO	0.51
MAR	0.27
MEX	0.07
MLT	0.78
MYS	0.01
NER	0.02
OMN	0.85
PAK	0.67
SAU	13.54
SOM	1.56
SVK	0.01
SYR	1.07

Continued on next page

Table S8 – continued from previous page

Country	β_0
TCD	0.02
THA	0.25
TKM	1.75
TTO	0.02
TUR	0.27
UGA	0.01
UKR	0.10
URY	0.08
USA	0.01
UZB	1.92
VEN	0.03
YEM	1.06
ZAF	0.53
ZWE	0.75

Supplementary References

1. Stefania Tamea, Marta Tuninetti, Irene Soligno, and Francesco Laio. Virtual water trade and water footprint of agricultural goods: the 1961–2016 cwasi database. *Earth System Science Data*, 13(5):2025–2051, 2021.
2. Marta Tuninetti, Stefania Tamea, Francesco Laio, and Luca Ridolfi. A fast track approach to deal with the temporal dimension of crop water footprint. *Environmental Research Letters*, 12(7):074010, 2017.
3. Marta Tuninetti, Luca Ridolfi, and Francesco Laio. Charting out the future agricultural trade and its impact on water resources. *Science of the Total Environment*, 714:136626, 2020. ISSN 0048-9697.
4. Marc François Müller, Jim Yoon, Steven M Gorelick, Nicolas Avisse, and Amaury Tilmant. Impact of the syrian refugee crisis on land use and transboundary freshwater resources. *Proceedings of the national academy of sciences*, 113(52): 14932–14937, 2016.
5. Jim Yoon, Christian Klassert, Philip Selby, Thibaut Lachaut, Stephen Knox, Nicolas Avisse, Julien Harou, Amaury Tilmant, Bernd Klauer, Daanish Mustafa, et al. A coupled human–natural system analysis of freshwater security under climate and population change. *Proceedings of the National Academy of Sciences*, 118(14), 2021.
6. Naota Hanasaki, Sayaka Yoshikawa, Yadu Pokhrel, and Shinjiro Kanae. A Quantitative Investigation of the Thresholds for Two Conventional Water Scarcity Indicators Using a State-of-the-Art Global Hydrological Model With Human Activities. *Water Resources Research*, 54(10):8279–8294, oct 2018. ISSN 0043-1397. . URL https://onlinelibrary.wiley.com/doi/abs/10.1029/2018WR022931.
7. Pardhasaradhi Teluguntla, Prasad S Thenkabail, Jun Xiong, Murali Krishna Gumma, Chandra Giri, Cristina Milesi, Mutlu Ozdogan, Russ Congalton, James Tilton, Temuulen Tsagaan Sankey, et al. *Land resources: monitoring, modelling, and mapping*, chapter Global Cropland Area Database (GCAD) derived from remote sensing in support of food security in the twenty-first century: current achievements and future possibilities. Taylor & Francis, 2015.
8. Nicole Jackson, Megan Konar, and Arjen Y Hoekstra. The water footprint of food aid. *Sustainability*, 7(6):6435–6456, 2015.
9. Mesfin M Mekonnen and Arjen Y Hoekstra. National water footprint accounts: the green, blue and grey water footprint of production and consumption. volume 2: appendices. *Daugherty Water for Food Global Institute: Faculty Publications*, 84, 2011.
