# Supplementary material for: Food demand displaced by global refugee migration influences water use in already water stressed countries
Source: Nat Commun. 2023 May 23;14:2706. doi: 10.1038/s41467-023-38117-0 (PMC10205736; doi:10.1038/s41467-023-38117-0)
Supplement: Supplementary file 3 — Reporting Summary [file 41467_2023_38117_MOESM3_ESM.pdf]

nature research

Corresponding author(s): Marc F. Muller

Last updated by author(s): Mar 7, 2023

Reporting Summary

Nature Research wishes to improve the reproducibility of the work that we publish. This form provides structure for consistency and transparency in reporting. For further information on Nature Research policies, see our Editorial Policies and the Editorial Policy Checklist.

Statistics

For all statistical analyses, confirm that the following items are present in the figure legend, table legend, main text, or Methods section.

n/a	Confirmed	
☐	☒	The exact sample size (*n*) for each experimental group/condition, given as a discrete number and unit of measurement
☒	☐	A statement on whether measurements were taken from distinct samples or whether the same sample was measured repeatedly
☐	☒	The statistical test(s) used AND whether they are one- or two-sided<br>*Only common tests should be described solely by name; describe more complex techniques in the Methods section.*
☐	☒	A description of all covariates tested
☐	☒	A description of any assumptions or corrections, such as tests of normality and adjustment for multiple comparisons
☐	☒	A full description of the statistical parameters including central tendency (e.g. means) or other basic estimates (e.g. regression coefficient) AND variation (e.g. standard deviation) or associated estimates of uncertainty (e.g. confidence intervals)
☐	☒	For null hypothesis testing, the test statistic (e.g. *F*, *t*, *r*) with confidence intervals, effect sizes, degrees of freedom and *P* value noted<br>*Give P values as exact values whenever suitable.*
☒	☐	For Bayesian analysis, information on the choice of priors and Markov chain Monte Carlo settings
☒	☐	For hierarchical and complex designs, identification of the appropriate level for tests and full reporting of outcomes
☒	☐	Estimates of effect sizes (e.g. Cohen's *d*, Pearson's *r*), indicating how they were calculated

Our web collection on statistics for biologists contains articles on many of the points above.

Software and code

Policy information about availability of computer code

Data collection	No software was used.
Data analysis	Data analysis was carried out using R v.4.1.0. and the following packages: fst (v.0.9.8), ggthemes (v.4.2.4), scales (v.1.2.1), weights (v. 1.0.4), plotly (v. 4.10.1), tidyverse (v. 1.3.2), boot (v. 1.3.28), ggplot2 (v. 3.4.0), countrycode (v. 1.4.0), and maps (v. 3.4.1) all of which are publicly available are no cost at \url{https://www.r-project.org}. The code developed to generate the figures is available at \url{https://doi.org/10.5281/zenodo.7779414}

For manuscripts utilizing custom algorithms or software that are central to the research but not yet described in published literature, software must be made available to editors and reviewers. We strongly encourage code deposition in a community repository (e.g. GitHub). See the Nature Research guidelines for submitting code & software for further information.

Data

Policy information about availability of data

All manuscripts must include a data availability statement. This statement should provide the following information, where applicable:

- Accession codes, unique identifiers, or web links for publicly available datasets
- A list of figures that have associated raw data
- A description of any restrictions on data availability

The per capita water footprint data generated in this study are available at \url{https://doi.org/10.5281/zenodo.7779414}. Refugee data were obtained from the UNHCR data platform \url{https://www.unhcr.org/refugee-statistics/}, the water footprint of primary and processed crops were obtained from the CWASI dataset \url{https://www.watertofood.org/download/}. All country-level water metrics were obtained from the FAO AQUASTAT database \url{https://www.fao.org/aquastat/en/}.

Field-specific reporting

Please select the one below that is the best fit for your research. If you are not sure, read the appropriate sections before making your selection.

☐ Life sciences ☒ Behavioural & social sciences ☐ Ecological, evolutionary & environmental sciences

For a reference copy of the document with all sections, see nature.com/documents/nr-reporting-summary-flat.pdf

Behavioural & social sciences study design

All studies must disclose on these points even when the disclosure is negative.

Study description	The study estimates the per capita water footprint of food by country, which is then used to estimate the country-level water stress induced by the food demand transferred by global refugee migration between 2005 and 2016. These are quantitative data.
Research sample	The sample includes all 167 countries or autonomous regions that were either the refuge or the origin (or both) of at least 1000 refugees during at least one year in the 2005-2016 period. The rationale for the inclusion criterion is that these countries meaningfully participate as either refugee origin or destination countries. Refugee data were obtained from the UNHCR data platform https://www.unhcr.org/refugee-statistics/
Sampling strategy	There is no sampling strategy: we analyze the full population of countries satisfying the inclusion criterion: hosting or providing at least 1000 refugees during at least one year of the 2005-2016 period.
Data collection	The analysis is based solely on published secondary data that are openly available on web-based repositories with hyperlinks given in the Methods section of the manuscript. No primary data collection was carried out: no instruments were used to record the data.
Timing	The analysis considers country-level annual data for the 2005-2016 period. The publicly available datasets listed in the data availability statement were accessed on September 15 2022.
Data exclusions	Twenty-nine of the 167 countries of the sample were removed from specific portions of the analysis due data incompleteness, as identified in Table S1 of supplementary information (also included in csv format in the data repository).
Non-participation	No participants dropped out.
Randomization	This is not relevant to our study because we analyzed the full population of countries (minus the data exclusions descriebed above) and did not carry out any randomization.

Reporting for specific materials, systems and methods

We require information from authors about some types of materials, experimental systems and methods used in many studies. Here, indicate whether each material, system or method listed is relevant to your study. If you are not sure if a list item applies to your research, read the appropriate section before selecting a response.

Materials & experimental systems

n/a	Involved in the study	
☒	☐	Antibodies
☒	☐	Eukaryotic cell lines
☒	☐	Palaeontology and archaeology
☒	☐	Animals and other organisms
☒	☐	Human research participants
☒	☐	Clinical data
☒	☐	Dual use research of concern

Methods

n/a	Involved in the study	
☒	☐	ChIP-seq
☒	☐	Flow cytometry
☒	☐	MRI-based neuroimaging
